# Novel RNAi-Based Therapies for Atherosclerosis

**DOI:** 10.1007/s11883-021-00938-z

**Published:** 2021-06-19

**Authors:** Anna-Kaisa Ruotsalainen, Petri Mäkinen, Seppo Ylä-Herttuala

**Affiliations:** 1 A.I. Virtanen Institute for Molecular Sciences, University of Eastern Finland, P.O. Box 1627, FIN-70211 Kuopio, Finland; 2 Kuopio Center for Gene and Cell Therapy, FIN-70210 Kuopio, Finland; 3 Heart Center and Gene Therapy Unit, Kuopio University Hospital, P.O. Box 1777, FIN-70211 Kuopio, Finland

**Keywords:** Atherosclerosis, Nucleic acid, RNAi, siRNA, Hypercholesterolemia, Hypertriglyceridemia

## Abstract

**Purpose of Review:**

Atherosclerosis, defined by inflammation and accumulation of cholesterol, extracellular matrix, and cell debris into the arteries is a common factor behind cardiovascular diseases (CVD), such as coronary artery disease, peripheral artery disease, and stroke. In this review, we discuss and describe novel RNA interference (RNAi)-based therapies in clinical trials and on the market.

**Recent Findings:**

The first RNAi-based therapies have entered clinical use for the control of atherosclerosis risk factors, i.e., blood cholesterol levels. The most advanced treatment is silencing of proprotein convertase subtilisin/kexin type 9 (PCSK9) with a drug called inclisiran, which has been approved for the treatment of hypercholesterolemia in late 2020, and results in a robust decrease in plasma cholesterol levels.

**Summary:**

As the new RNAi therapies for atherosclerosis are now entering markets, the usefulness of these therapies will be further evaluated in larger patient cohorts. Thus, it remains to be seen how fast, effectively and eminently these new drugs consolidate their niche within the cardiovascular disease drug palette.

## Introduction

RNA interference (RNAi) is an evolutionary mechanism of silencing gene expression in most eukaryotic cells. In RNAi, complementary double-stranded RNA molecules called small interfering RNAs (siRNAs) are directed to cleave the specific sequence of the target mRNA. As two decades has elapsed from the initial discovery that RNA-mediated gene silencing through RNAi appears also in mammalian cells [1], novel drug products based on this phenomena have cleared their ways to the market. The first-in-human clinical trial of an RNAi therapy was performed in cancer patients eight years ago [2]. However, by the end of 2020, only three RNAi drugs had been approved by both European Medicines Agency (EMA) and Food and Drug Administration (FDA) in the USA [3, 4]. These first drugs have been targeted against genes related to rare genetic disorders. However, they provide a proof-of-concept for the effectiveness of RNAi therapies and open new treatment possibilities also for more common diseases like dyslipidemias. Multiple RNAi-based therapeutics are now entering for clinical trials also for cardiovascular diseases (CVD) and recently, one therapy has been approved for clinical use. In addition, several antisense oligonucleotide (ASO)-based treatments are potential for CVD therapies, from which the first one was approved for clinical use in 2019. Like siRNAs, ASOs also bind to the complementary target mRNA sequence and prevent protein translation, but ASOs are single-stranded RNA or DNA molecules. A remarkable milestone in the development of liver targeted RNAi and ASO therapies has been the *N*-acetylgalactosamine (GalNAc) delivery technology, that has improved the tissue specificity of therapeutic conjugate molecules [5, 6]. Hepatocytes are expressing high levels of asialglycoprotein receptors, in which GalNAc-conjugated molecules are bind and efficiently endocytosed into the liver.

Atherosclerotic CVD still remain one of the leading causes of death worldwide. As hypercholesterolemia is one of the most potent risk factors for CVD, therapies directed against lowering lipoprotein levels in circulation are eagerly awaited. One of the novel targets aiming at lipid lowering is the downregulation of proprotein convertase subtilisin/kexin type 9 (PCSK9). PCSK9 regulates cholesterol homeostasis by binding to low-density lipoprotein receptor (LDLR) and it has been a popular target for therapeutic interventions with antibodies and other small molecule compounds [7, 8]. Likewise, RNAi-based therapies against PCSK9 have been developed and will be discussed in more detail below. In addition to PCSK9, other new promising therapeutic targets have entered clinical trials (Table 1) and are also coming to the hyperlipidemia market. One of the most promising one for the treatment of hyperlipidemias is angiopoietin-like 3 (ANGPTL3) which regulates the activity of lipoprotein (LPL) and endothelial lipases (EL) in peripheral tissues. ANGPTL3 function is proposed to be independent of LDLR activity, and it has several functions in the liver by regulating the hydrolysis of triglycerides (TGs), uptake of low-density lipoprotein (LDL) and very low-density lipoprotein (VLDL) particles, and VLDL secretion [9]. However, RNAi-mediated ANGPTL3 silencing had only minor effect on plasma low-density lipoprotein cholesterol (LDL-C) in the presence of LDLR-deficiency in mice, but in wild-type mice, the reduction in plasma LDL-C, high-density lipoprotein cholesterol (HDL-C) and TG levels were significant. This is also supported by add-on effect in combination of ANGPTL3 and PCSK9 siRNAs [10]. In addition, apolipoprotein CIII (APOCIII) has similar functions with ANGPTL3 and it is an independent risk factor for CVD. APOCIII is found in chylomicrons, VLDL, and remnant particles, and it is recognized as a potential therapeutic target for hyperlipidemia since ApoC-III loss-of-function mutations have been reported to lead to low plasma TG levels and reduced risk for CVDs [11•]. Finally, one of the most significant risk factors for atherosclerotic CVD is an atherothrombotic LDL-like plasma lipoprotein (a) [Lp(a)]. It has several proatherogenic and thrombogenic functions and it contains oxidized phospholipids capable of activating monocytes, inducing foam cell formation, regulating platelet activity, and promoting endothelial dysfunction [12]. Elevated Lp(a) levels have been found in familial hypercholesterolemia (FH) and it is associated with coronary heart disease in large studies.

**Table 1:** Overview of approved and ongoing RNAi-based clinical trials

Target	Drug name	Phase	Approach	Main outcome	Trial no.	Reference(s)
PCSK9	Inclisiran	Approved	GalNAc-siRNA	Up to ~ 53% reduction in LDL-C. No serious adverse effects.	NCT03399370, NCT03400800, NCT03397121	16,17
ANGPTL3	ARO-ANG	Phase I/II	GalNAc-siRNA	LDL-C of 39–42% in 22 hypercholesteraemic patients, and mean maximum reductions in TG of 79%	NCT03747224	24
APOCIII	ARO-APOCIII	Phase I/II	GalNAc-siRNA	Reduction in TG up to 70% and elevation in plasma HDL up to 80%	NCT03783377	28
Lp(a)	Olpasiran	Phase II	GalNAc-siRNA	Reduction in plasma Lp(a) levels up to 94%	NCT03626662 NCT04270760	40

This review focuses on recent RNAi-based therapies for atherosclerotic CVDs, which have advanced into clinical development during the past 3 years.

### PCSK9

Inclisiran is an GalNAc-modified siRNA, which targets PCSK9, and therefore decreases the expression of PCSK9 mRNA by RNAi. PCSK9 inhibitors reduce the degradation of transmembrane LDL Rs and increase the uptake of LDL, thus lowering plasma LDL-C levels. As inflammation plays a key role in atherogenesis, reduction of PCSK9 is reported to decrease the expression of vascular chemokines and adhesion molecules [13]. Phase II trials have already provided encouraging results of the performance of inclisiran in patients with atherosclerotic cardiovascular disease and familial hypercholesterolemia [14, 15] and three phase III trials have also been completed with inclisiran. ORION-10 trial (NCT03399370) was conducted with 1561 patients with established atherosclerotic cardiovascular disease, and ORION-11 (NCT03400800) trial enrolled 1617 patients with atherosclerotic cardiovascular disease equivalent or high primary prevention [16]. Third completed trial is ORION-9 (NCT03397121) with 482 heterozygous FH patients [17••].

In all of these above-mentioned studies, 300 mg doses of inclisiran sodium (or corresponding 284 mg of inclisiran free acid) on days 1, 90, 270 and 450 were used as subcutaneous 1.5 mL injections. A recent meta-analysis concerning these three studies has summarized the main results [18]. The analysis confirms that LDL cholesterol is decreased 51% compared to the placebo group [18]. In addition, decrease in total cholesterol was 37%, apolipoprotein B 41%, and non-HDL-C 45% [18]. Overall, these decreases were associated with a 24% decrease in cardiovascular events, such as cardiac death, cardiac arrest, myocardial infarction, or stroke. Thus, it seems that inclisiran is very effective in LDL reduction, and it creates a long-lasting LDL lowering effect. Only semiannual injections are enough to maintain the effect.

Importantly, no serious adverse effects were reported in these three trials, but some mild injection site reactions occurred [18]. A recent publication from phase 1 ORION-1 trial shows that inclisiran does not exert adverse effects on platelet counts, and has no effect on lymphocyte, monocyte, or neutrophil counts at day 180 [19]. Some patients have been followed up to 360 days without significant side effects. Also, inclisiran has been proven to be safe in patients with renal impartment, and no dose adjustment for those patients is needed [20]. Therefore, it looks like inclisiran is not only efficient against its primary target leading to favorable lipoprotein alterations in patients but is also accompanied with a really good safety profile. Overall, the success of inclisiran gives confidence on siRNA-directed RNAi therapies and proves that a long-term therapeutic effect can be achieved with injectable medicinal formulations. inclisiran has been already accepted by EMA in late 2020, and the drug is entering European markets named as Leqvio® [21]. FDA has not approved inclisiran yet, not due to safety issues, but due to COVID-19-related travel restrictions and further inspection delays on manufacturing site [21].

### ANGPTL3

The first ANGPTL3 inhibitor, monoclonal antibody evinacumab, has recently approved as an add-on treatment for adult and pediatric FH patients. Evinacumab decreases plasma LDL-C levels 49% and TG levels up to 75% [22] and is well tolerated. Also, other technologies, like ASO have been used to inhibit ANGPTL3 expression. The efficiency on plasma TG lowering was promising, as ASO against ANGPTL3 decreases TG levels up to 63% in humans [23]. In addition, this GalNAc-conjugated ASO called IONIS-ANGPTL3-L_Rx_ was well tolerated. In addition to hypertriglyceridemia, this therapy is promising also for the treatment of type 2 diabetes mellitus and non-alcoholic fatty liver disease, in which phase II clinical trial is ongoing (NCT03371355). Monoclonal antibodies have specific effects on circulating target molecules whereas RNAi enables the inhibition of intracellular gene expression. ANGPTL3-targeted siRNA, ARO-ANG3, is studied for the treatment of heterozygote FH patients, and is currently in phase I clinical trial (NCT03747224). It is considered as a potential treatment also for hyperchylomicronemia and hypertriglyceridemia syndromes. In FH patients, ARO-ANG3 therapy in combination with statins reduced plasma LDL-C levels up to 37% and TG up to 43% [24••]. In non-FH patients, the reduction of plasma LDL-C and TG was equal in FH patients. Subcutaneously injected ARO-ANG3 has been well tolerated, but some respiratory tract infections (30% of subjects) and injection site adverse effects (13% of subjects) were reported. ANGPTL3 inhibition therapy gives new hope for the treatment of hyperlipidemias and atherosclerotic CVDs. However, it is notable that in addition to reduction in plasma remnant lipoprotein levels, ANGPTL inhibition reduces plasma HDL levels. In a phase 3 trial (Evinacumab Lipid Studies in Patients with Homozygous Familial Hypercholesterolemia, ELIPSE HoFH), evinacumab reduced HDL cholesterol by 30% [25]. So, it is a concern that ANGPTL3 inhibition reduces HDL-C cholesterol levels, while reducing LDL levels, and thus may not be an ideal treatment for HoFH to reduce progression of atherosclerosis and cardiovascular complications.

### ApoCIII

ApoC-III has similar effects with ANGPTL as it regulates TG hydrolysis by inhibiting LPL activity. ApoC-III is present in chylomicrons, VLDL, and remnant particles, and its loss-of-function mutations have been reported to lead to low plasma TG levels and reduced risk for CVDs [11•]. In addition to the regulation of plasma lipoproteins, APOC-III has several other mechanisms that have a direct impact on the development of atherosclerotic plaques. ApoC-III regulates the accumulation and aggregation of proatherogenic lipoproteins with the arterial wall proteoglycans by modulating the composition of the lipoprotein and ApoB conformation [11•]. Moreover, APOC-III enhances the activation of vascular adhesion molecules and inflammatory cytokine pathways, and promotes migration and adhesion of circulating monocytes which leads to increased accumulation of inflammatory cells in the vascular wall [26, 27].

RNAi with ARO-APOC-III modestly reduced plasma LDL-C, but caused a significant reduction in plasma TG levels up to 70%, and elevated plasma HDL levels by ~ 80% in healthy volunteers [28••]. The therapy was well tolerated, although some injection site adverse effects and headaches were reported. RNAi selectively suppresses APOC-III expression with good tolerability and has potential for treating patients with chylomicronemia at risk of pancreatitis.

Other therapeutic approaches have been also developed to inhibit ApoC-III expression. The first approved ApoC-III ASO, volanesorsen, was approved by FDA in 2019 [29]. A phase II study showed reduction in plasma TGs [30] and VLDL levels [31] in patients with hypertriglycemia. In addition, in phase III studies with familial chylomicronemia syndrome and hypertriglyceridemia (NCT02211209, NCT02300233), TG levels were reduced > 70% [32, 33]. However, injection site reactions were common side effects [29, 33]. Several ApoC-III targeted ASOs have been developed and are currently in clinical trials. GalNAc-conjugated ASO against ApoC-III and AKCEA-APO-CIII-L_Rx_ are promising, as those decreased TG levels on average up to 77% in a phase I/IIa study in healthy volunteers and were well tolerated [34]. It will be interesting to see whether RNAi-based approaches exceed the efficacy and safety of ApoC-III ASOs.

### Lp(a)

Another interesting target and an individual risk factor for atherosclerosis is lipoprotein (a) [Lp(a)] [35]. It has been suggested that Lp(a) could serve as an additional screening and treatment target similar to traditional LDL-centered therapies [36]. Moreover, a recent meta-analysis has shown that statin therapy might actually increase Lp(a) levels [37•], whereas PCSK9 inhibitor evolocumab has reported to reduce Lp(a) plasma levels ~ 25% [38]. Phase II trial with ASOs have been shown to effectively decrease Lp(a) levels [39]. Also, A GalNAc-modified siRNA targeting Lp(a), named AMG 890 or olpasiran, is currently tested in phase I and phase II trials (NCT03626662, NCT04270760). The first results have been published in a conference abstract, and olpasiran seemed to decrease Lp(a) 80–94% at day 113. No imminent adverse effects were observed [40]. As the early results are promising, more widespread Lp(a) screenings and clinical results are waited for, so that the role of Lp(a) lowering as a therapeutic target can be better assessed.

### Advantages and Challenges of RNAi-Based Therapeutics in Lipid Lowering

The current and forthcoming siRNA-based therapeutics have shown promising and long-lasting results in lipid lowering and reduction of CVD risk. As lipid metabolism and atherogenesis are both regulated by several complex pathways, new potential targets and applications for therapeutic interventions are under investigation. However, the combinations of current therapies are worth of noticing, as many statin treated patients reach improved efficiency on plasma lipid lowering with PCSK9 inhibition. In addition to plasma LDL-C lowering, the reduction of plasma VLDL and remnant lipoprotein levels significantly reduce the risk for CVD [41, 42]. Thus, the targeting of different pathways of lipid metabolism and atherogenesis, like inflammatory pathways, may give great benefit in the reduction of CVD risk. Interestingly, the triple combination of alirocumab and evinacumab with statin treatment in hyperlipidemic mouse model, showed great reduction in plasma TG, total cholesterol, and non-HDL-C in comparison to single therapies [43]. This study also showed the total inhibition of atherogenesis, regression of atherosclerotic plaques, and even the beneficial effect on plaque morphology and stability in mouse model. However, the specific mechanisms of combination treatments still require further investigation.

## Conclusions

In summary, multiple RNAi-based therapeutics are now entering for phase II and III trials for CVD and already one new therapy has been approved for clinical use. In addition to inclisiran, it appears that ANGPTL3, APOCIII, and Lp(a)-targeted therapies are very attractive candidates that protect against atherosclerosis, and a whole new avenue will be launched in the treatment of CVD risk factors with RNAi-based medicines in the near future.
